# Overexpression of thymic stromal lymphopoietin is correlated with poor prognosis in epithelial ovarian carcinoma

**DOI:** 10.1042/BSR20190116

**Published:** 2019-05-14

**Authors:** Lu Xu, Yacong Guo, Ning Xu, Lihai Chen, Jin Zhu, Ningsheng Liu, Zhi-Yuan Zhang

**Affiliations:** 1 Department of Pathology, Nanjing Medical University, Nanjing 210000, China; 2 Department of Anesthesiology, Nanjing First Hospital, Nanjing Medical University, Nanjing 210000, China; 3 Huadong Medical Institute of Biotechniques, Nanjing 210002, China; 4 The Key laboratory of Antibody Technique of Ministry of Health, Nanjing Medical University, Nanjing 210000, China

**Keywords:** epithelial ovarian cancer, immunohistochemistry, prognosis, thymic stromal lymphopoietin

## Abstract

Thymic stromal lymphopoietin (TSLP) is an IL-7-like cytokine that has been reported to be associated with several malignant tumors. The present study aimed to evaluate its role in epithelial ovarian carcinoma (EOC). The mRNA levels of TSLP in human EOC samples and EOC cell lines were determined. Then, the expression of TSLP was examined in 144 clinical tissue microarray samples and correlated with clinicopathological factors. Finally, the correlation between TSLP overexpression and prognosis of EOC patients was analyzed. Our data show that mRNA levels of TSLP were significantly higher in EOC tissues and cell lines. Chi-square tests revealed that TSLP overexpression in EOC was significantly associated with age, histological type, Federation of Gynecology and Obstetrics (FIGO) stage, histological differentiation, pelvic involvement, and lymphatic metastasis. Kaplan–Meier survival analysis revealed that poor prognosis was significantly correlated with older age, advanced FIGO stage, poor histological differentiation, pelvic involvement, lymphatic involvement, or TSLP overexpression (*P*<0.05). Additionally, multivariate Cox regression analysis confirmed pelvic involvement and TSLP overexpression as independent prognostic factors for both overall and disease-free survival. Taken altogether, TSLP overexpression reflects a more malignant phenotype and TSLP may be a novel biomarker for EOC.

## Introduction

Ovarian carcinoma is common amongst women and is one of the most lethal carcinomas of the genital system. Epithelial ovarian cancers (EOC) are the most common serous carcinoma and account for the majority of ovarian malignancies [1]. Approxiately 22,440 new cases of ovarian cancer are reported each year in the U.S.A., and this disease was expected to cause over 14,070 deaths in 2018 [1]. Furthermore, approximately 52,100 new cases of ovarian cancer and 22,500 associated deaths are reported each year in China [2]. Since more than two-thirds of ovarian cancer cases are diagnosed at advanced stages (stage III–IV), a high mortality rate for this disease is observed [3]. Despite improvements in surgery, chemotherapy, and targeted drugs, the 5-year survival rate (<30%) for late-stage cases has remained unchanged since the 1980s, compared with >70% for early-stage cases [4]. Since only a few early-stage ovarian carcinomas can be diagnosed, it is difficult to predict the disease progression using standard clinical or pathological prognostic factors, such as grade, stage, or expressions of CA125/MUC16. Therefore, to the development of novel molecular biomarkers to improve the diagnosis and prognosis prediction for ovarian carcinoma is urgently required.

Thymic stromal lymphopoietin (TSLP) is an IL-7-like cytokine that had been initially identified by its ability to regulate inflammatory responses at the barrier surfaces [5–8]. TSLP is now considered to have multiple effects on different immune cells including T cells, B cells, and dendritic cells, which are involved in tumor initiation, growth, angiogenesis, metastasis, and tumor immunity [9–13]. Therefore, TSLP may play a role in cancer progression. Recent studies have suggested that TSLP contributes to the development of malignant tumors such as Hodgkin disease, pancreatic, cervical, breast, and gastric cancers [14–19]. TSLP is essential for maintaining Th2-type homeostasis [20,21], which has been reported in allergic diseases [22] and tumors [23,24]. Patients with cancers in which Th2-type responses predominate usually have poorer prognosis than the ones in which Th1-type responses predominate [14]. Interestingly, Th2- type responses have been observed during the development of ovarian carcinoma [25]. We, therefore, assumed that TSLP might be involved in the ovarian carcinoma and could serve as a biomarker or as a therapeutic target. In the present study, we investigated mRNA levels in two well-known ovarian cancer cell lines and examined expression patterns of TSLP in a large amount of tissue samples obtained from EOC patients. Moreover, we analyzed the correlation between TSLP overexpression and clinicopathological characteristics, to clarify its potential prognostic value in EOC patients.

## Materials and methods

### Tissue samples

Ovarian cancer samples (*n*=27) and the adjacent normal counterparts were collected from 2013 to 2015 at Nanjing Maternity and Child Health Care Hospital affiliated of Nanjing Medical University and with the approval of the Nanjing Medical University Ethics Committee. Samples were stored in liquid nitrogen for RNA extraction.

### Cell lines and reagents

The human EOC cell lines, SKOV3 and HO8910 [26] derived from ovarian adenocarcinoma and the immortalized ovarian epithelial cell line IOSE386 [27] preserved in our laboratory, were used in this study. Cells were maintained in Dulbecco’s modified Eagle’s medium (Gibco, Waltham, MA, U.S.A.) containing 10% FBS, and 1% penicillin/streptomycin.

### RNA isolation and RT-PCR

The ovarian cancer tissue samples were cut into 1 mm^3^, lysed and then RNA was extracted with Eastep^®^ Super Total RNA Extraction Kit (Promega, Madison, WI, U.S.A.). RNA of EOC cell lines was extracted with TRIzol (Invitrogen) according to the manufacturer’s instruction. PrimeScript Reverse Transcriptase (Takara, Dalian, China) was used to reverse RNA to cDNA. The relative expression of TSLP was detected with ChamQ SYBR qPCR Master Mix (Vazyme, Nanjing, China) on the LightCycler 96 (Roche, Basel, Switzerland). The specific primers were as follows: for TSLP (F: 5′-CCCAGGCTATTCGGAAACTCAG-3′and R: 5′-CGCCACAATCCTTGTAAT TGTG-3′) and GAPDH (F: 5′-GGAAGGTGAAGGTCGGAGTCA-3′ and R: 5′- GTCATTGA TGGCAACAATATCCACT-3′).

### Clinical information and preparation of tissue microarrays

Tissue microarrays were provided by Nantong University Hospital with approval of the local Ethics Committee. For details, 144 patients with primary EOCs of different histological types, 25 benign diseases, 38 borderline ovarian tumours, 25 normal ovaries, and 29 fallopian tubes were collected from patients who underwent surgery between April 2004 and September 2009 at Nantong University Hospital, Nantong, China. Ovarian carcinomas were defined by a two-tier (negative/low expression and overexpression) system. The record of patients reviewed in the context of clinicopathological information including age, surgical procedure, histological type, tumor stage, histological grade, presence of ascites, pelvic involvement and lymph node involvement, CA199, CA125, CA153, SF, menopause, survival time, and survival status. The overall survival (OS) and disease-free survival (DFS) were employed for survival analysis. OS was defined as the period from initial diagnosis to the date of death or last follow-up visit. DFS was defined as the time interval between the date of surgery and the date of disease recurrence. The records of patients, who were alive at follow-up or who did not die of disease, were censored. None of the patients had received preoperative radiotherapy or chemotherapy. Complete follow-up data until May 2016 were documented in all these cases.

To prepare the tissue microarrays, the representative cancer area was labeled in specific paraffin blocks according to hematoxylin and eosin staining results. A tissue array needle was inserted to obtain a 2-mm-diameter tissue sample, with one core for each sample. The tissue was then sequentially aligned into the prepared blank paraffin blocks. The tissue microarrays were cut into 4 μm sections and placed on tissue microarray-specific adhesive-coated glass slides. All tissues were reviewed by two gynecological pathologists to verify the diagnosis, histological grade, and stages. Pathological stage and histological subtype were determined for each surgical specimen according to 2013 International Federation of Gynecology and Obstetrics (FIGO) criteria and WHO classification.

### Immunohistochemistry

After routine deparaffinization, rehydration, blocking with hydrogen peroxide, and tissue antigen retrieval with a microwave, the tissue microarrays were incubated with rabbit polyclonal antibody to TSLP (GTX85060, 1:500, GeneTex, Irvine, California, U.S.A.), stained with secondary antibody (Dako REAL EnVision Detection System, Dako, U.K.), counterstained with hematoxylin and then evaluated independently by two pathologists who were unaware of the clinical parameters. Discordant cases were re-evaluated on a double-headed microscope to achieve a consensus. Expression levels of TSLP protein were evaluated by observing the incidence and staining intensity of immunohistochemically positive cells, as described by Abubaker [28]. Briefly, the IHC staining intensity was scored as 0 (negative staining), 1 (weak staining), 2 (moderate staining), and 3 (strong staining), and the percentage of positive cells defined as 0 = 0%, 1 ≤ 10%, 2 = 10–50%, 3 = 51–80%, and 4 ≥ 80% cells. Multiplying intensity and percentage provided an overall expression score and a cutoff of 4 was used to define the overexpression of TSLP.

### Statistical analysis

TSLP mRNA levels of tissue samples and cell lines were evaluated with the Mann–Whitney U test. The Chi-square test was used to analysis expression of TSLP protein in tissue microarrays. To determine their prognostic significance, the Cox proportional hazards model was applied to assess clinicopathological parameters for univariate and multivariate analyses. Survival curves were constructed with the Kaplan–Meier method, and the differences between the curves were compared by the log-rank test. When *P*<0.05, the difference was considered significant. The statistical analyses were performed with the SPSS software package (SPSS Standard version 16.0, SPSS Inc., Chicago, IL, U.S.A.).

## Results

### Evaluation of TSLP mRNA levels in EOC tissue samples and EOC cell lines

**Figure 1:**
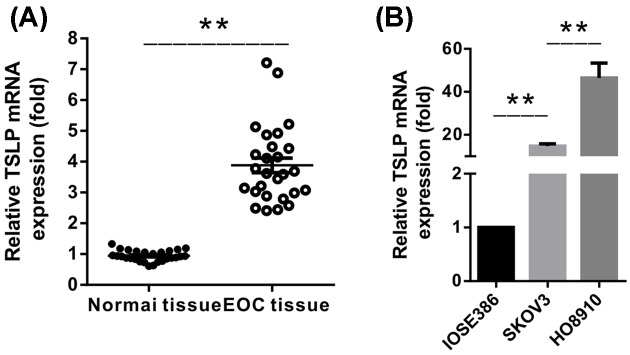
The mRNA levels of TSLP in EOC (**A**) The mRNA levels of TSLP were significant higher in EOC tissue samples. (**B**) The mRNA levels of TSLP were enhanced in EOC cell lines. (**P<0.01).

Compared with adjacent normal tissues, TSLP mRNA levels determined by real-time PCR were significantly higher in human ovarian cancer tissues (*n*=27), (*P*<0.001) (Figure 1A), implying the potential function of TSLP in ovarian cancer. Meanwhile, significantly elevated mRNA levels of TSLP were detected in widely recognized EOC cell lines SKOV3 and HO8910, in comparison with ovarian epithelial cell line IOSE386 (Figure 1B). Our results showed that TSLP was overexpressed in EOC and suggested that it might be involved in tumor biology or development of EOC.

### Expression of TSLP in EOC microarray

**Figure 2:**
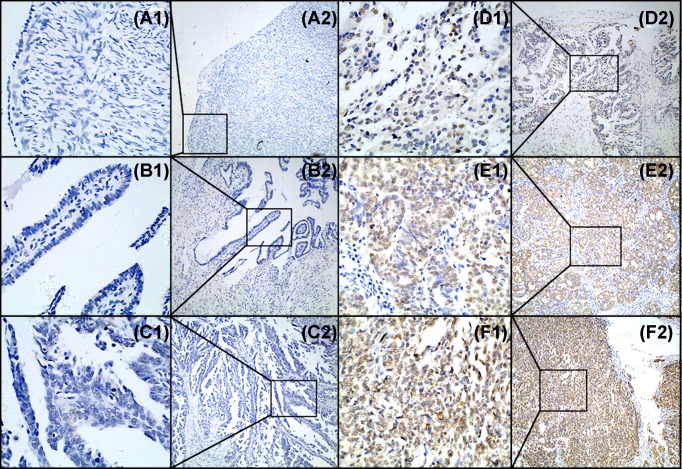
The expression pattern of TSLP was determined by IHC in tissue microarrays (**A**) Negative expression of TSLP in normal ovarian epithelium sample (score 0). (**B**) Negative expression of TSLP in normal fallopian tube sample (score 0). (**C**) Negative expression of TSLP in EOC tissue (score 0). (**D**) Low expression of TSLP in EOC tissue (score 4). (**E,F**) Overexpression of TSLP in EOC tissues (E: score 10 and F: score 12). Original magnification ×400 in A1, B1, C1, D1, E1, and F1; ×100 in A2, B2, C2, D2, E2, and F2.

TSLP expression patterns were further evaluated in 144 primary EOC tissue microarrays. As shown in Figure 2 and Table 1, higher expression of TSLP was observed in 85 from total 144 patients with EOC (59%), while TSLP overexpression was only detected in four benign cases (16%) and nine borderline cases (24%). In control tissues, overexpression of TSLP was only observed in the normal ovarian/fallopian tubes for 11 of 54 cases (20.4%). Our IHC results revealed that overexpression of TSLP was detected in EOC tissues, compared with control tissues from ovaries/fallopian tubes, benign cases, and borderline cases (*P*<0.001).

**Table 1:** TSLP overexpression of normal ovaries/fallopian tubes, benign, borderline, and malignant carcinomas

	Total patients (*n*)	TSLP overexpression		*P-*value
		No *n* (%)	Yes *n* (%)	
Normal (a)	54	43 (79.6%)	11 (20.4%)	0.645 (a/b)
				0.704 (a/c)
				<0.001*(a/d)
Benign (b)	25	21 (84%)	4 (16%)	0.461(b/c)
				<0.001*(b/d)
Borderline (c)	38	29 (76.3%)	9 (23.6%)	<0.001*(c/d)
Malignant (d)	144	59 (40.9%)	85 (59.1%)	

The *P* values were calculated by Chi-square test.

* *P*<0.05 was considered significant.

### Clinicopathological characteristics of patients with EOC

In our present study, we also collected the clinical features and clinicopathological factors of the EOC patients. Table 2 shows that 144 patients with primary EOC had a median age of 53.9 years (range: 24–79 years) at diagnosis. Amongst 144 patients, 89 (61.4%) showed serous EOC, 7 (4.8%) with mucinous EOC, 13 cases (9.0%) with endometrioid EOC, ten cases (6.9%) of clear cell EOC and mixed EOC respectively, seven cases (4.8%) involved transitional cell EOC, and eight cases (5.5%) involved undifferentiated EOC. The FIGO stages were stage I for 73 patients, stage II for 8 patients, stage III for 62 patients, and stage IV for 1 patient. Out of 144, 34 cases had good differentiation, and the rest have moderate or poor differentiation. A total of 76 patients exhibited pelvic involvement, and 25 cases were complicated by lymph node metastasis. Ascites was detected in 51 patients and 90 cases involved menopausal women.

**Table 2:** Patients’ characteristics and correlation of patients’ clinicopathological parameters with TSLP overexpression

	Total patients (*n*)	TSLP overexpression		*P-*value*
		No *n* (%)	Yes *n* (%)	
*Age at diagnosis(y)*				
<60	89	44 (49.4%)	45 (50.6%)	0.009*
≥60	55	15 (27.3%)	40 (72.7%)	
*Histological type*				
Serous	89	33 (37.1%)	56 (62.9%)	0.022*
Mucinous	7	5 (71.4%)	2 (28.6%)	
Endometriosis	13	6 (46.2%)	7 (53.8%)	
Clear cell	10	5 (50%)	5 (50%)	
Mixed	10	8 (80%)	2 (20%)	
Transitional cell	7	1 (14.3%)	6 (85.7%)	
Undifferentiated	8	1 (12.5%)	7 (87.5%)	
*FIGO stage*				
I and II	81	46 (56.1%)	35 (43.9%)	<0.001*
III and IV	63	13 (22.2%)	50 (77.8%)	
*Histological differentiation*				
G1	34	20 (58.8%)	14 (41.2%)	0.015*
G2	110	39 (35.5%)	71 (64.5%)	
*Pelvic involvement*				
No	77	46 (59.7%)	31 (40.3%)	<0.001*
Yes	67	13 (19.4%)	54 (80.6%)	
*Lymph node involvement*				
No	110	52 (47.3%)	58 (52.7%)	0.034*
Yes	25	6 (24%)	19 (76%)	
No data	9			
*Presence of ascites*				
No	93	41 (44.1%)	52 (55.9%)	0.305
Yes	51	18 (35.3%)	33 (64.7%)	
*Menopause*				
No	53	29 (54.7%)	24 (45.2%)	0.080
Yes	90	29 (32.3%)	61 (67.7%)	
No data	1			
*CA199*				
Negative	78	23 (29.5)	55 (71.5)	0.064
Positive	24	12 (50%)	12 (50%)	
No data	42			
*CA125*				
Negative	13	5 (38.5%)	8 (61.5%)	0.856
Positive	92	33 (35.9%)	59 (64.1%)	
No data	39			
*CA153*				
Negative	42	18 (42.9%)	24 (57.1%)	0.123
Positive	54	15 (27.8%)	39 (72.2%)	
No data	48			
*SF*				
Negative	68	28 (41.2%)	40 (58.8%)	0.089
Positive	30	7 (23.3%)	23 (76.7%)	
No data	46			

G1: low-grade serous, low-grade mucinous, both grades I and II endometrioid, mixed and transitional cell EOCs;

G2: high-grade serous, high-grade mucinous, clear cell, undifferentiated EOCs and grade III endometrioid, mixed and transitional cell EOCs.

*P* values were calculated by Chi-square test.

* *P*<0.05 was considered significant.

### Relationships between TSLP overexpression and clinicopathological characteristics

As shown in Table 2, the relationship between TSLP overexpression and the patient’s clinicopathological characteristics was analyzed. Overexpression of TSLP in EOC was significantly associated with age (*P*=0.009), histological type (*P*=0.022), FIGO stage (*P*<0.001), histological differentiation (*P*=0.015), pelvic involvement (*P*<0.001), and lymphatic metastasis (*P*=0.034). No other significant associations were observed between TSLP overexpression and other clinicopathological characteristics.

### Survival analysis

**Figure 3:**
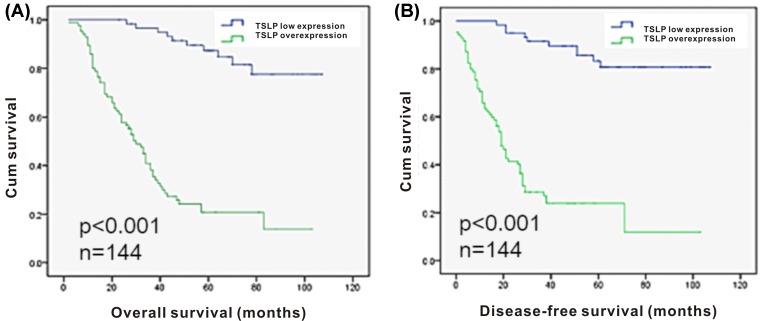
Survival analysis of EOC patients by the Kaplan–Meier method (**A**) Kaplan–Meier survival curve for overall survival of patients and TSLP overexpression (*P*<0.001). (**B**) Kaplan–Meier survival curve for disease-free survival of patients and TSLP overexpression (*P*<0.001).

A total of 144 cases were followed until May 2016 for the survival analysis. During the follow-up process, 72 patients had died, with a median overall survival (OS) of 38.4 months and a median disease-free survival (DFS) of 29.1 months. Compared with the low TSLP expression group, the overexpression group had significantly lower OS and DFS values. Table 3 shows the associations of OS and DFS with age at diagnosis, advanced FIGO stage, poor histological differentiation, pelvic involvement, lymph node involvement, and TSLP overexpression. The multivariate Cox proportional hazards models revealed that poor survival was associated with TSLP overexpression and pelvic involvement, respectively. Kaplan–Meier survival analysis revealed shorter OS and DFS amongst patients with TSLP overexpression (Figure 3).

**Table 3:** Univariate and multivariate analysis (Cox regression model) of OS and DFS in relation to various parameters and TSLP overexpression

Parameter	OS			DFS		
	Univariate^#^	Multivariate^##^		Univariate^#^	Multivariate^##^	
	*P* value	*P* value	Risk ratio (95% CI)	*P* value	*P* value	Risk ratio (95% CI)
TSLP IHC	<0.001*	<0.001*	7.308	<0.001*	<0.001*	7.399
Low(-)/Over(+)			(3.538–15.09)			(3.507–15.61)
Age, y	0.001*	0.150	1.428	0.001*	0.115	1.486
<60/≥60			(0.879–2.321)			(0.908–2.432)
FIGO stage	<0.001*	0.834	1.069	0.000*	0.725	1.117
<IIIC//≥IIIC			(0.573–1.992)			(0.602–2.074)
Histologic differentiation	0.006*	0.495	1.331	0.005*	0.917	0.962
Well/Mod and poorly			(0.585–3.027)			(0.465–1.990)
Pelvic involvement	<0.001*	<0.001*	3.718	0.000*	0.003*	2.731
No/Yes			(1.837–7.526)			(1.396–5.343)
Lymph node involvement	0.010*	0.210	1.416	0.009*	0.202	1.428
No/Yes			(0.822–2.438)			(0.826–2.468)
Presence of ascites	0.078			0.086		
No/Yes						
Menopause		0.106		0.093		
No/Yes						

TSLP IHC Low expression: total score 0–4; TSLP IHC Overexpression: total score 5–12.

OS: the time between the date of initial diagnosis and death or last contact;

DFS: the time between the date of surgery and identification of disease recurrence;

CI: confidence interval.

# Statistical analyses were performed by log-rank test.

## Statistical analyses were performed by Cox regression model.

* *P*<0.05 was considered significant.

## Discussion

Ovarian carcinoma is known for its high mortality rate (75%) and difficulty in diagnosis, especially at the early stage. Further, the prognosis of ovarian carcinoma is not properly predicted with usual clinical and pathological parameters, and only limited prognosis markers are presently available. Therefore, the development of novel and more efficient molecular biomarkers will be helpful to improve the diagnosis, prognosis prediction, and targetted therapeutics for ovarian carcinoma. In the present study, we discovered significantly increased mRNA levels of TSLP in EOC cell lines and tissues, and significantly higher TSLP expression in EOC tissues. In addition, the correlation of TSLP overexpression with various clinicopathological factors, such as pathological differentiation, FIGO stage, pelvic, and lymph node metastases, was found. Furthermore, Kaplan–Meier survival curve analysis revealed significantly shorter OS and DFS of patients with TSLP overexpression; this suggested that TSLP overexpression may serve as a marker of poor prognosis in patients with EOC.

As a member of the IL-2 cytokine family and a distant paralog of IL-7 [6], oncogene properties of TSLP, such as pro-tumorigenic, cancer cell protective, and tumor growth promoting properties, have been reported in various animal models of cancers [17,29–31], as well as in studies conducted on humans. TSLP overexpression was detected in breast cancer cells [16] and cancerous tissues of Hodgkin disease, and cervical and gastric cancer [15,19]s. Genetic rearrangements and mutations in the TSLP gene were detected in lymphoblastic leukemia [32–34]. TSLP released from human cervical carcinoma cells promoted angiogenesis and cancer growth [24], while the blockage of TSLP suppressed tumor growth and infiltration [16,23,24].

Recent studies have indicated that TSLP overexpression may promote the survival, growth, and metastasis of many solid tumors with a Th2-biased immune response, including cervical, breast and pancreatic cancer, and cutaneous T-cell lymphoma [7,14–17]. Th2-biased immune responses are crucial in tumor growth and development [23], and cancer patients with Th2 infiltration usually have a poor prognosis than those with a Th1-biased or CD8^+^ response. Experiments conducted on TSLP receptor-deficient mice indicated that TSLP might play an essential role in protection and metastasis of cancer cells [28] through its receptor on CD4^+^T cells. TSLP induces the production of immunosuppressive factors such as IL-10, IL-13, and chemokines, which recruit suppressive immune cells and promote Th2-biased immune responses. This helps malignant cells to escape from immune surveillance and protects them from immune attack [29]. Moreover, the deficiency of TSLP in cancer cells alone can efficiently abrogate cancer progression and lung metastasis [17].

Ovarian carcinoma has also been identified as a Th2-biased micro-environment [22]. Further, ovarian cancers were suppressed by targeting Th2-biased inflammatory response, such as IL-4R-targeted therapies [23,24] or by using therapies that shift the immunosuppressive Th2 inflammation to the immunostimulatory Th1 type [35]. Concurrent with these previous reports, our results revealed significant univariate associations of TSLP overexpression with FIGO grade, histological differentiation, pelvic involvement, and lymph node involvement in EOC; thus, indicating aggravated cancer growth, progression, and metastasis. Further, increased expression of TSLP was observed during the progression of the EOC tumors from benign or borderline and finally to malignant. Since TSLP overexpression could drive the development of Th2-dominant carcinomas, our results here suggest that TSLP may also play an important role in growth, invasion, and progression of EOC, possibly through manipulation of the immune response.

Kaplan–Meier analysis revealed worse OS and DFS outcomes amongst patients with TSLP overexpression, compared with patients with negative or low TSLP expression. Multivariate Cox regression analysis confirmed TSLP overexpression and pelvic involvement as independent prognostic factors for both overall and disease-free survival. Notably, the prognostic value of pelvic involvement has been long recognized in most cancers of female genital system. Our statistical analyses showed that TSLP overexpression could be used as a prognostic marker and indicator of poor prognosis in ovarian cancer patients.

All our findings suggest that TSLP overexpression could facilitate the growth and metastasis of EOC cells, promote a Th2-biased microenvironment and may subsequently lead to poor prognosis. Nonetheless, more evidences are needed to test these hypotheses. In addition, these results suggest the pathological role of TSLP in the development of other malignant carcinomas, as well as the poor prognosis of patients with TSLP overexpression.

In conclusion, this is the first study revealing the correlation of TSLP overexpression in EOC patients with cancer progression and shorter survival time. These data suggest that TSLP might be a useful biomarker to predict the prognosis in EOC patients and the development of anti-TSLP therapy for EOC merits further investigation.

## Abbreviations

DFS: disease-free survival
EOC: epithelial ovarian cancer
FIGO: Federation of Gynecology and Obstetrics
IHC: immunohistochemistry
OS: overall survival
TSLP: thymic stromal lymphopoietin
